# Potassium silicate and vinasse enhance biometric characteristics of perennial sweet pepper (*Capsicum annuum*) under greenhouse conditions

**DOI:** 10.1038/s41598-024-61454-z

**Published:** 2024-05-16

**Authors:** Mahmoud S. Rady, Ibrahim M. Ghoneim, Mostafa N. Feleafel, Shimaa M. Hassan

**Affiliations:** 1 Department of Vegetable Crops, Faculty of Agriculture (El-Shatby), Alexandria University, Alexandria, 21545 Egypt; 2 Plant and Environmental Sciences Department, Coastal Research and Education Center, Clemson University, Charleston, SC 29414 USA

**Keywords:** *Capsicum annuum*, Sweet pepper, Potassium silicate, Vinasse, Condensed molasses soluble, Greenhouse, Agroecology, Plant development, Plant ecology, Plant physiology, Plant reproduction, Plant stress responses

## Abstract

An effective strategy for enhancing fruit production continuity during extended sweet pepper season involves adopting innovative biostimulants such as potassium silicate (PS) and vinasse. Adjusting PS and vinasse concentrations are crucial for maintaining the balance between vegetative and fruit growth, particularly in sweet pepper with a shallow root system, to sustain fruiting over prolonged season. However, the interaction between PS and vinasse and the underlying physiological mechanisms that extend the sweet pepper season under greenhouse conditions remain unclear. This study aimed to investigate the impact of PS and vinasse treatments on the yield and biochemical constituents of perennial pepper plants cultivated under greenhouse conditions. For two consecutive seasons [2018/2019 and 2019/2020], pepper plants were sprayed with PS (0, 0.5, and 1 g/l) and drenched with vinasse (0, 1, 2, and 3 l/m^3^). To estimate the impact of PS and vinasse on the growth, yield, and biochemical constituents of pepper plants, fresh and dry biomass, potential fruit yield, and some biochemical constituents were evaluated. Results revealed that PS (0.5 g/l) coupled with vinasse (3 l/m^3^) generated the most remarkable enhancement, in terms of plant biomass, total leaf area, total yield, and fruit weight during both growing seasons. The implementation of vinasse at 3 l/m^3^ with PS at 0.5 and 1 g/l demonstrated the most pronounced augmentation in leaf contents (chlorophyll index, nitrogen and potassium), alongside improved fruit quality, including total soluble solid and ascorbic acid contents, of extended sweet pepper season. By implementing the optimal combination of PS and vinasse, growers can significantly enhance the biomass production while maintaining a balance in fruiting, thereby maximizing the prolonged fruit production of superior sweet pepper under greenhouse conditions.

## Introduction

Sweet pepper (*Capsicum annuum* L.) holds significant economic importance as a widely cultivated vegetable crop in Egypt and around the world^1^. The widespread popularity of sweet pepper can be attributed to its high contents of ascorbic acid and essential elements such as potassium, iron, and magnesium. These nutrients not only enhance overall human health but also reinforce immune system functions^2^. Additionally, sweet pepper fruit contain various carotenoids, involving oxygenated carotenoids and β-carotene, which play a crucial function in preventing the development of common illnesses such as cataracts, diabetes, and cancer^3^. Egypt contributes approximately 680,000 tons to the global sweet pepper production of 37 million tons, placing it the sixth worldwide^4^. Noteworthy, sweet pepper holds the second rank (33%) among vegetables cultivated under greenhouse conditions in Egypt, following cucumber. Furthermore, the cultivation area of greenhouse sweet pepper in Egypt covers approximately 9.68 million m^2^, with a yield of around 8.193 kg/m^2^^5^. These statistics underscore the significant role of sweet pepper among Egypt’s most valuable greenhouse vegetable.

Addressing the challenges presented by growing populations and changing weather patterns requires substantial effort to achieve and maintain global food security^6^. An essential aspect of this endeavor involves adopting innovative agricultural management practices that can yield vegetables consistently and in an environmentally friendly manner. These practices aim to enhance the vigor of vegetables, a critical factor in ensuring optimal growth, yield, and fruit quality^7^. Recently, there has been a growing interest in the use of new biostimulants like potassium silicate (PS) and vinasse as cost-effective and practical tools for sustainable crop management in agriculture^8^.

Potassium silicate (PS) serves as a source of soluble silicon and potassium, containing approximately 34.5% SiO_2_ and 25% K_2_O^9^. Although silicon (Si) is not considered an essential plant nutrient, it is recognized as a beneficial element that enhances plant metabolic processes in a hormetic manner. Treatment of Si on pepper plant has been discovered to enhance the levels of phenolic compounds in fruit cell walls. These phenolic polymers act as a barrier, enhancing the cell walls’ resilience against mechanical and enzymatic degradation^10^. Silicon also serves multiple functions in plant physiology, such as regulating K uptake, increasing carotenoids and chlorophyll contents, and reducing water stress by decreasing transpiration through stomatal closure^11,12^. By modulating physiological processes, Si exhibits biostimulant effects on plant, promoting growth, development, and stress responses^13,14^. Moreover, K is an essential macronutrient for elevating photosynthetic rates through enhanced CO_2_ assimilation, participates in protein synthesis, and augments levels of photosynthetic pigments. Potassium also plays a pivotal role in facilitating carbohydrate translocation from the shoot to the fruit. The combined action of Si and K actively mitigate oxidative stress while regulating stomatal activity and controlling transpiration rates^12,15^, thereby enhancing both plant growth, physiological processes, and productivity of sweet pepper^11^.

Vinasse, also known as condensed molasses soluble, is a significant byproduct of the fermentation industry. It finds applications in various sectors, including food production, distilleries, sugar manufacturing, and yeast production. However, these processes result in substantial volumes of water-containing waste^16^. In recent years, vinasse has gained attention due to its high content of organic material and natural mineral nutrients. Its utilization not only enhances agricultural production but also addresses the issue of wastewater disposal^17^. In contrast, excessive use of chemical fertilizers can negatively impact soil chemistry, physics, and biology, leading to environmental pollution^18^. Vinasse, with its acidic properties and abundant organic and soluble compounds, can serve as a fertilizer for cash crops^19^. Additionally, vinasse enhances the availability of essential macro and micronutrients and organic matter in the soil, ultimately improving plant growth and production^20^. Moreover, vinasse promotes growth and photosynthetic rates without detrimental effects. Substituting mineral fertilizers with vinasse contributes to resource recycling, a key aspect of sustainable horticultural production^21^.

Sweet pepper, typically grown as an annual crop due to its susceptibility to cold weather, exhibits herbaceous perennial traits and can continuously produce new stems, leaves, flowers, and fruits for over a year under suitable temperature conditions^22^. Consequently, greenhouse conditions play a vital role in the cultivation of winter-spring vegetable, extending the sweet pepper growing season to 9–10 months^23,24^. Previous studies have demonstrated the short-term positive impacts of PS foliar application on water use efficiency, stress tolerance, plant growth and yield of vegetables such as pepper^25,26^, tomato^27,28^, potato^29^, mustard^30^, and chicory^31^, as well as the vinasse effects on the growth and productivity of field crop such as sugarcane, maize, wheat^32,33^, and leafy greens i.e. cabbage and lettuce^34,35^. However, the prolonged impact on fruity vegetables grown for over a year under greenhouse conditions, specifically sweet pepper, remains relatively unexplored. Therefore, our study seeks to address this gap by examining the long-term effects of PS, vinasse, and their synergy on the plant growth and fruit productivity of sweet pepper, with a particular focus on extending the fruiting season beyond typical annual cycles. By demonstrating, for the first time, how the synergistic effect of PS and vinasse can extend the fruiting season of sweet peppers for over a year, this novel approach not only contributes to our understanding of sustainable agricultural practices but also holds potential implications for enhancing food security and resource efficiency in greenhouse sweet pepper production. Consequently, we hypothesize that: (i) the combined application of PS and vinasse will improve the balance between vegetative growth and fruit development in sweet pepper by enhancing nutrient uptake and photosynthesis, thus boosting plant productivity; (ii) the synergistic effects of PS and vinasse will improve the fruit quality; and (iii) the application of optimal concentrations of PS and vinasse will promote plant growth during the extended growing season, ultimately leading to an increased relative yield.

The main objective of this study was to maintain plant vigor and a balance between leaf growth and fruit bearing in sweet pepper plant for over a year. This was achieved through improved management practices, including the application of vinasse through drenching and PS through foliar spray. The study aimed to investigate how these practices impact the developmental biometric traits, productivity, and fruit quality of greenhouse-grown sweet pepper plant.

## Material and methods

This experiment was conducted at the Experimental Station Farm of the Faculty of Agriculture, Alexandria University, Egypt (31°12'48.4" N, 29°59'06.6" E), during the seasons spanning from 2018 to 2019 and from 2019 to 2020. The aim was to investigate the impact of different concentrations of PS and vinasse on various biometric characteristics and crop productivity of sweet pepper plants (*Capsicum annuum* L.) grown under a plastic quonset greenhouse (area of 240 m^2^), comprising 3 raised beds each with 2 m width and 40 m length.

Prior to the experiment, soil samples were randomly collected from the greenhouse at a depth of 15 to 30 cm. These samples were analyzed at the Unit of Analysis and Scientific Services, Alexandria University, following the methods described by^36^ to assess various chemical and physical properties.

### Experimental treatments

The experimental treatments involved applying different concentrations of PS (K_2_SiO_3_) as a foliar spray (0, 0.5, and 1 g/l) on four occasions, spaced at intervals of 15, 30, 45, and 60 days following the start of the season (DFSS), which commenced on September 20th, 2018, and September 20th, 2019, for the first and second seasons, respectively. Additionally, four concentrations of vinasse (0, 1, 2, and 3 l/m^3^ water) were applied weekly as a drench at 0.25 l per plant, starting from 14 DFSS and continuing for nine weeks. Namely, throughout each individual season, plants received varying total amounts of concentrated vinasse, including 0, 5, 10, and 15 cm^3^/m^2^ soil. The PS was obtained from El-Gomhouria Company for Drugs, Egypt, while the vinasse was analyzed at the Bio-Tech for Biocides and Fertilizers Company, Egypt. The trial design utilized a randomized complete blocks design (RCBD), including three replicates per treatment, following a split-plot system. The main plots were arranged randomly for PS concentrations, while the subplots were randomly distributed for vinasse concentrations.

The greenhouse was equipped with a drip irrigation system that included lateral pipes measuring 16 mm in width and extending 40 m in length. These pipes were outfitted with emitters positioned every 0.5 m, totaling 80 emitters per line. Each raised bed was assigned two of these lateral pipes. Each emitter was designed to drip water at 4 l/h. Before transplanting, the irrigation system was activated for approximately 110 minutes to deliver an initial water irrigation of 3.5 m^3^. Sweet pepper hybrid ‘Marvel F1 cv.’ seeds were purchased from Nongwoo Bio Company, Suwon, Gyeonggi-do, South Korea. On September 20th, 2018, sweet pepper seedlings, with four fully grown leaves, were transplanted into the soil with a spacing of 40 cm between each plant, 100 plants per row, and 50 cm between two rows on each bed. Watering occurred every three days with 800 ml of water per plant in the morning.

**Figure 1:**
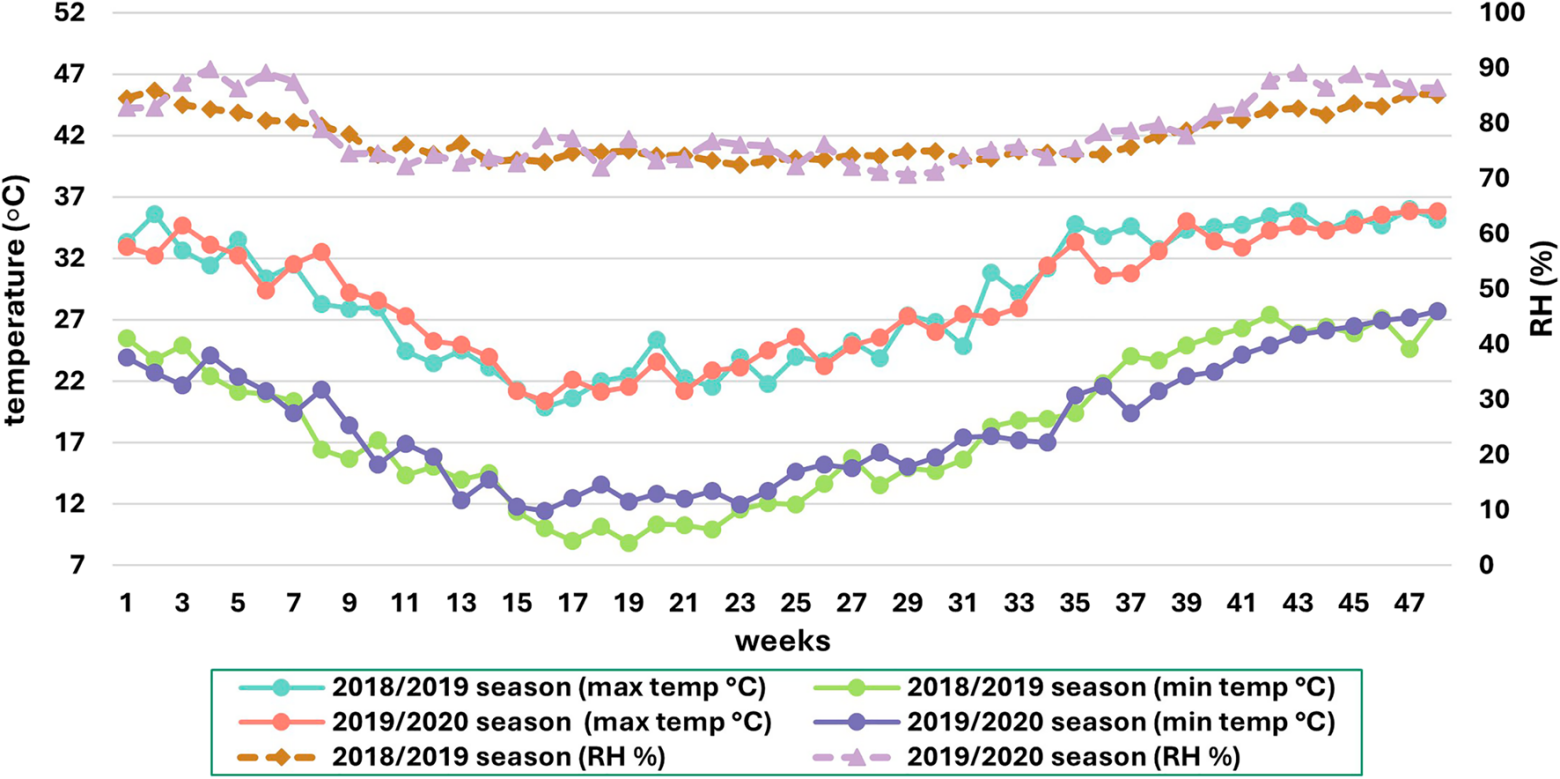
The greenhouse microclimatic conditions, including minimum and maximum air temperature (°C) and relative humidity (RH%), during the 2018/2019 and 2019/2020 seasons.

At the end of the first season on August 20th, 2019, plants were pruned, leaving a 30 cm stub above the soil to promote initial vegetative growth as the second season commenced on September 20th, 2019. The treatments from the previous season were replicated. The greenhouse microclimate, including relative humidity (RH%) and air temperature (°C), was monitored using the Testo 175-H1 sensor, TESTO Company, USA, as presented in Fig. 1. Additionally, cultural practices, including fertilization, irrigation, cultivation techniques, as well as disease and pest control measures, were implemented according to the recommended guidelines for commercial sweet pepper production under greenhouse conditions^37,38^. These practices were adjusted whenever necessary to maintain optimal growing conditions and plant health throughout the experimental period. The sweet pepper plant collection and use was in accordance with all the relevant guidelines and regulations.

### Measurements and calculations

#### Vegetative characteristics

At 120 DFSS, four random plants were selected from each subplot to measure their fresh and dry weight during both years. Dry biomass of different plant parts was determined by drying 100 g of fresh samples from various organs at 70°C until a constant weight was achieved. The dried plant material weight was recorded in grams per individual plant^39^. Leaf area per plant (cm^2^) was calculated based on the weight of fresh leaves. Ten leaves were randomly chosen from the selected plants, and their leaf segments were used to estimate leaf area following the method outlined by^40^.

$$\text{Leaf area }({{\text{cm}}}^{2}/{\text{plant}}) =\frac{\text {area of }10 \text { known leaf segments}*\text {leaf blades weight of }4 \text { plants }}{\text {weight of the same }10 \text{ segments}*4}$$

#### Harvest quantity of fruit and their components

Green-stage mature fruit were harvested in the morning up to 280 DFSS. For each harvest, twenty fruit were randomly chosen to determine the average weight (in grams) of fresh fruit. The mean value of all harvested fruit was computed at the end of the season. Moreover, at the end of the fruiting period, the total fruit harvest was assessed by measuring the weight per square meter. Additionally, the calculation for the percentage of relative yield was performed as follows:

$$\mathrm{Relative}\,\mathrm{yield}(\mathrm{\%}) =\frac{\mathrm{Fruit}\, \mathrm{yield}\left({{\text{kg}}/{\text{m}}}^{2}\right)\mathrm{of} \, \mathrm{a}\, \mathrm{specific} \, \mathrm{treatment}}{\mathrm{Fruit}\, \mathrm{ yield}\left({{\text{kg}}/{\text{m}}}^{2}\right)\mathrm{of}\, \mathrm{control }\, \mathrm{treatment}*}*100$$

^*^The control treatment refers to 0 g/l of PS and 0 l/m^3^ of vinasse.

#### Composition of leaf and fruit components

Fresh leaves were randomly sampled from four sweet pepper plants within each subplot at 120 DFSS. This was done to assess the relative chlorophyll content, in addition to the levels of nitrogen (N) and potassium (K) in the leaves. The Minolta SPAD chlorophyll meter model (SPAD-502 plus, Konica Minolta, Japan) was used to measure the greenness of the initial fully grown leaves by quantifying their relative chlorophyll content using the SPAD index. This measurement method was carried out as per the described technique^41^. Nitrogen (N) and potassium (K) levels were determined in dehydrated leaves obtained from the aforementioned plants. Before analysis, samples were oven-dried for 48 hours at 70°C until reaching a constant weight, ground through 1 mm mesh, and homogenized^42^. The leaves were milled, and a 0.3 g sample underwent digestion using hydrogen peroxide. The approach outlined by^43^ was employed to determine the total N and K contents.

Moreover, at 90 DFSS, five random fruits at the green stage were collected from each experimental unit to analyze the concentration of total soluble solids (TSS) and ascorbic acid compounds in the fruit flesh juice. The percentage of TSS was evaluated using a portable digital refractometer called Refractometer-Pal-1 from Japan. Additionally, the content of ascorbic acid was estimated through a titration process involving 2,6-dichloroindophenol, following the method described by^36^.

#### Statistical analysis

Statistical analysis was performed on all acquired data using analysis of variance (ANOVA) for each of the two growing seasons, in line with^44^. Co-Stat software (Co-Hort 6.303, Monterey, CA, USA)^45^ was utilized for the analysis. The revised least significant difference (LSD) test was applied to make comparisons between the means of different treatments. A significance level of 0.05 (with *P* ≤ 0.05) was established. Figures were generated using Python version 3.10 and the Google Colaboratory web interface.

## Results

### Plant biomass and growth characteristics:

**Figure 2:**
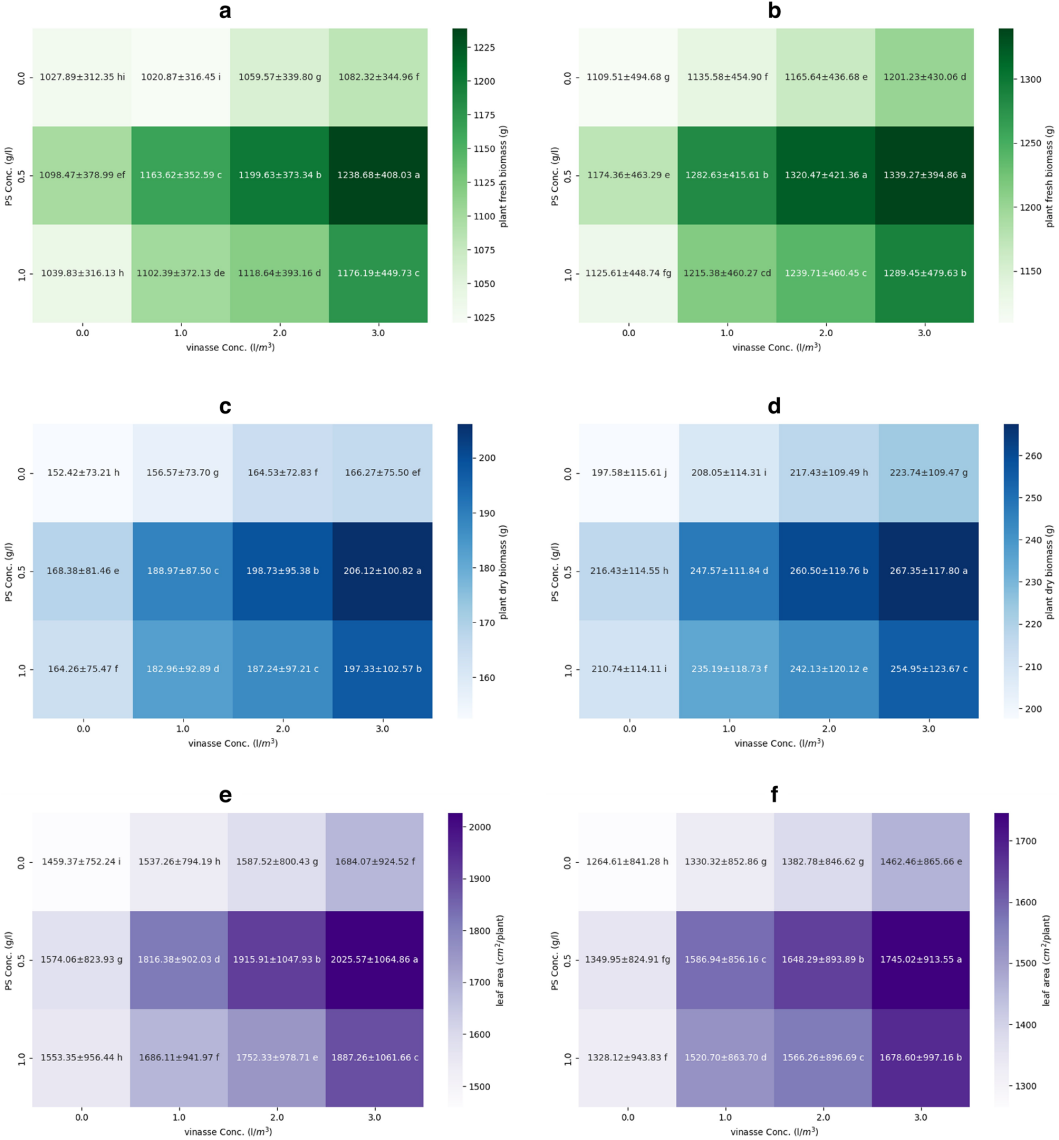
Effects of potassium silicate (PS) and vinasse concentrations interactions on plant fresh biomass (**a, b**), plant dry biomass (**c, d**), and total leaf area per plant (**e, f**) of sweet pepper plants during the 2018/2019 and 2019/2020 seasons. Values are means ± standard error (SE) (*n* = 3). In the same season, means followed by the same alphabetic letter(s), do not differ significantly, according to the LSD test at a probability level of 0.05.

As shown in Fig. 2, there was a consistent increase in all parameters -fresh plant biomass, dry plant biomass, and individual plant leaf area- for all PS concentrations (0, 0.5, and 1 g/l) across both growing seasons as vinasse concentration increased from 0 to 3 l/m^3^. Significant peak values were observed for all vegetative characteristics (plant biomass and leaf area per plant) when vinasse (3 l/m^3^) was combined with PS (0.5 g/l). Specifically, plant fresh biomass exhibited 1.21 and 1.20 times higher values compared to the lower responses obtained from vinasse (1 l/m^3^) with PS (0 g/l) and vinasse (0 l/m^3^) with PS (0 g/l) in the initial and subsequent seasons, respectively (Fig. 2a,b). Similarly, plant dry biomass exhibited 1.35 times higher value (Fig. 2c,d), while leaf area per plant showed 1.39 and 1.38 times higher value (Fig. 2e,f) when treated with vinasse (3 l/m^3^) in conjunction with PS (0.5 g/l) compared to vinasse (0 l/m^3^) with PS (0 g/l) in the initial and subsequent seasons, respectively. Furthermore, PS (0.5 g/l), when combined with any vinasse concentration, consistently demonstrated superior performance across all vegetative characteristics. Notably, while plant fresh and dry biomass generally exhibited higher values compared to the initial season, leaf area per plant was lower in the second season. Our findings indicate that high levels of vinasse with PS (0.5 g/l) have a significant beneficial influence on plant growth.

### Fruit yield characteristics

**Figure 3:**
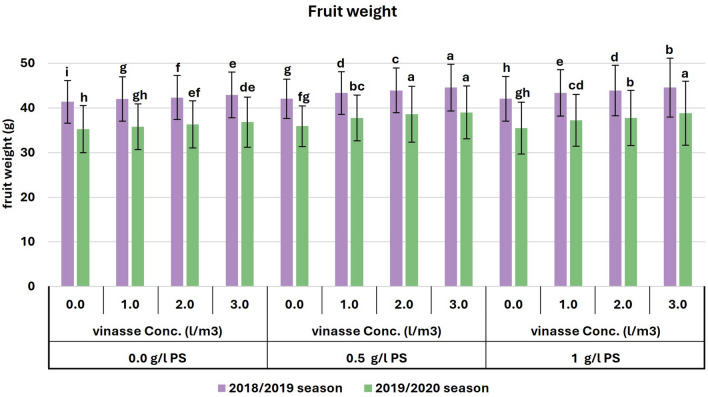
Effects of PS and vinasse concentrations interactions on the fruit weight of sweet pepper plants during the 2018/2019 and 2019/2020 seasons. Error bars showed standard error as the means ± SE (*n* = 3). In the same season, means followed by the same alphabetic letter(s), do not differ significantly, according to the LSD test at a probability level of 0.05.

**Figure 4:**
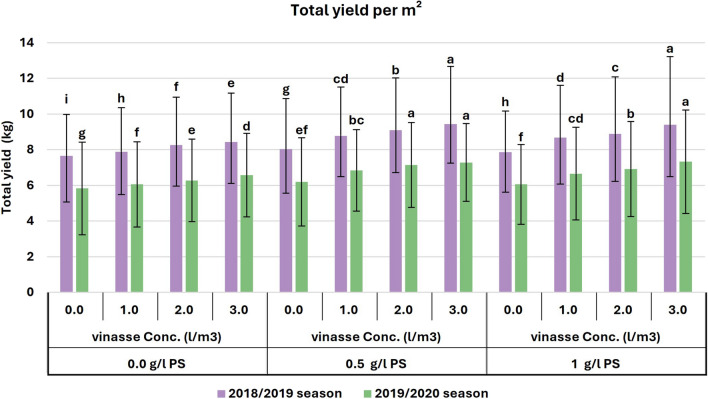
Effects of PS and vinasse concentrations interactions on the total yield per m^2^ of sweet pepper plants during the 2018/2019 and 2019/2020 seasons. Error bars showed standard error as the means ± SE (*n* = 3). In the same season, means followed by the same alphabetic letter(s), do not differ significantly, according to the LSD test at a probability level of 0.05.

**Figure 5:**
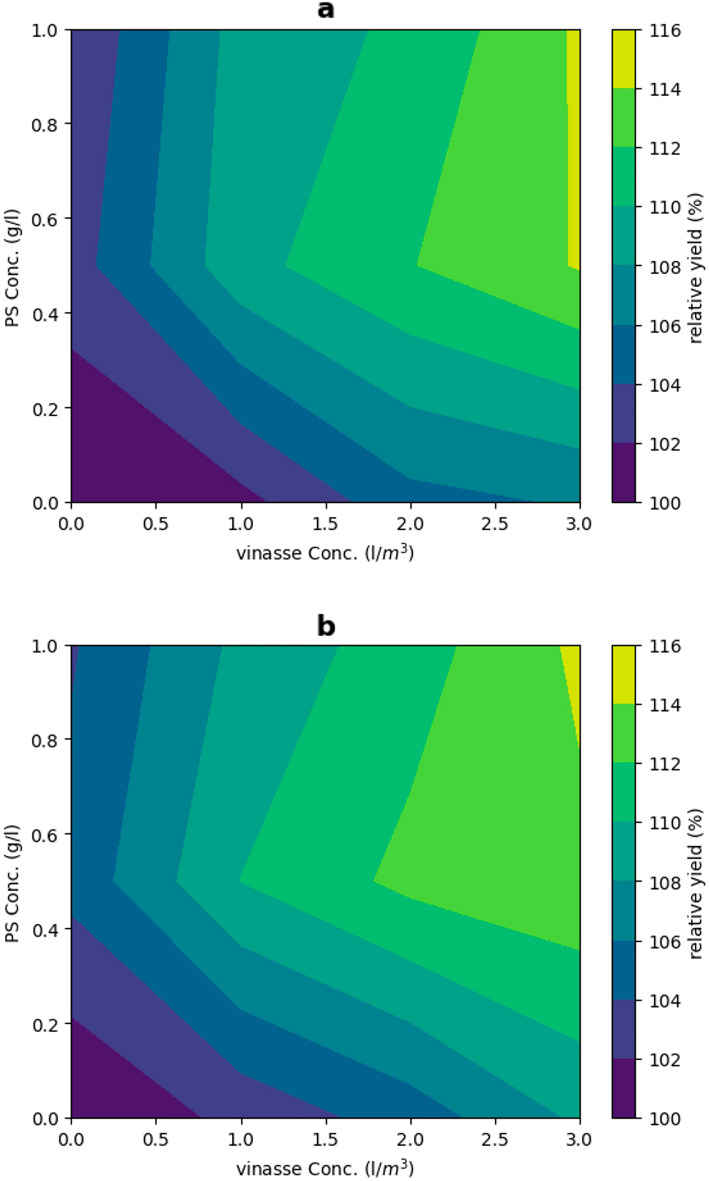
Interaction effect between PS and vinasse concentrations on the relative yield of sweet pepper plants during the 2018/2019 (**a**) and 2019/2020 (**b**) seasons.

In terms of fruit weight, the greatest average measurement was observed during the initial season when foliar treatment of 0.5 g/l PS was combined with drenching vinasse (3 l/m^3^). Similarly, during the second season, the best average result was achieved by using PS (0.5 g/l) along with applying 2 or 3 l/m^3^ of drenching vinasse, as well as with adding PS (1 g/l) along with drenching vinasse at 3 l/m^3^ (Fig. 3). Furthermore, the total yield per m^2^ significantly peaked with drenching vinasse at 3 l/m^3^ combined with PS (0.5 and 1 g/l) in both seasons. A significant peak at 7.14 kg/m^2^ was observed with vinasse (2 l/m^3^) combined with PS (0.5 g/l) in the second season (Fig. 4). The relative yield gradually increased with increasing PS and vinasse concentrations in both seasons. The interaction between PS and vinasse had a more pronounced impact on relative yield in the second season compared to the first season. Relative yield superior to 110% was achieved with approximately 0.3 g/l PS and 1.3 l/m^3^ of vinasse in the first season (Fig. 5a), and with approximately 0.2 g/l PS and 1 l/m^3^ of vinasse in the second season (Fig. 5b).

### Chemical components of leaves and fruit

**Figure 6:**
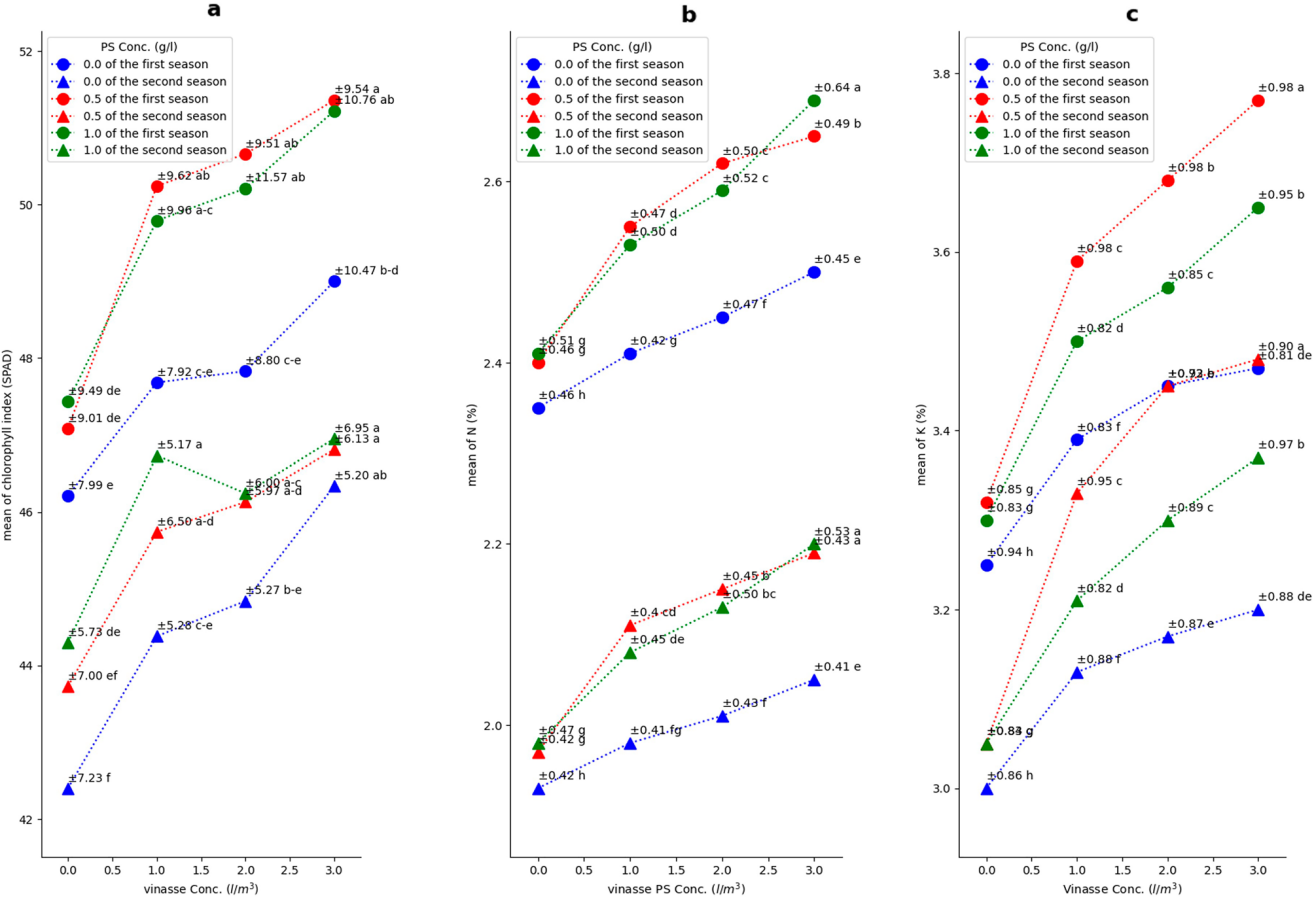
Effect of PS and vinasse concentrations interactions on leaves’ chlorophyll index (**a**), N (**b**), and K contents (**c**) of sweet pepper plants during the 2018/2019 and 2019/2020 seasons. Values are means ± (SE) (*n* = 3). In the same season, means followed by the same alphabetic letter(s), do not differ significantly, according to the LSD test at a probability level of 0.05.

**Figure 7:**
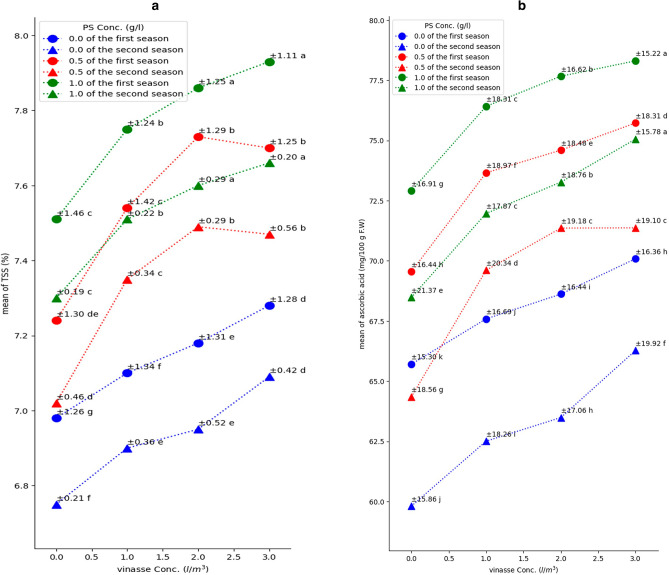
Effect of PS and vinasse concentrations interactions on fruits’ TSS (**a**) and ascorbic acid contents (**b**) of sweet pepper plants during the 2018/2019 and 2019/2020 seasons. Values are means ± (SE) (*n* = 3). In the same season, means followed by the same alphabetic letter(s), do not differ significantly, according to the LSD test at a probability level of 0.05. F.W. means fresh weight of fruit.

The results revealed significant and gradual increases in leaf chlorophyll index and N and K contents with increasing vinasse concentration from 0 to 3 l/m^3^ under all PS concentrations in both seasons. Notably, the combination of vinasse drenching at 1, 2, or 3 l/m^3^ with the foliar application of PS (0.5 or 1 g/l) in both seasons, as well as vinasse (3 l/m^3^) with PS (0 g/l) in the second season, resulted in the most substantial average chlorophyll index in sweet pepper leaves (Fig. 6a). The most notable increase for leaf N content was achieved with vinasse (3 l/m^3^) along with PS (1 g/l) in the initial season and with PS either 0.5 or 1 g/l in the subsequent season (Fig. 6b). Similarly, drenching vinasse at 3 l/m^3^ in the first season and at 2 or 3 l/m^3^ in the second season, combined with PS (0.5 g/l), exhibited the greatest significant leaf K content (Fig. 6c). In terms of fruit constituents, increasing vinasse concentration from 0 to 3 l/m^3^ with any PS concentration led to significant and successive increment in TSS and ascorbic acid within sweet pepper fruit in both growing seasons (Fig. 7). The most significant fruit TSS was achieved by applying 2 or 3 l/m^3^ of vinasse along with PS (1 g/l) in both seasons (Fig. 7a). Furthermore, applying the vinasse at 3 l/m^3^ along with applying PS (1 g/l) during both growing seasons led to a notable rise in fruit ascorbic acid level, compared to the other treatments employed (Fig. 7b).

## Discussion

The observed positive effects on sweet pepper plant’s biomass and growth attributes due to drenching vinasse with varying PS concentrations could be attributed to changes in soil osmotic pressure^46–48^ , potentially leading to increased water and nutrient uptake, thus affecting metabolic activities like photosynthesis and respiration, influencing plant growth^49^. The presence of potassium in PS and vinasse could enhance carbon assimilation and sugar transport^15,50^, while silicon may boost photosynthesis by increasing chlorophyll concentration and affecting the activities of RuBisCO and PEP-carboxylase enzymes for CO_2_ fixation^51,52^. Vinasse is rich in both major and trace nutrients (Table 2), which play pivotal role in various physiological processes and promote plant vigor. Particularly, the ample N content in vinasse significantly boosts nitrogen assimilation in photosynthetic parts, thus competing for energy compounds and the carbon pool generated through photosynthesis^53^. This fostered cell division, elongation, and increased leaf area for growth^54^. Adequate K also plays a critical role in essential plant processes such as photosynthesis, transmembrane transport, activation of numerous biocatalysts, protein synthesis, and facilitation of cell division and growth by aiding in carbohydrate movement within different plant sections^55^. Additionally, K is implicated in plant’s response to abiotic stress, regulating plant stomata and water usage, especially in dry environments^56^, much like the conditions described in the ongoing research. Furthermore, the observed positive effects on plant biomass resulting from vinasse drenching could potentially arise from the limited presence of essential nutrients, specifically N, K, and Mg in the soil used in the experiments, as indicated in Table 1. The recorded nutrient concentrations in the experimental soil were 0.462% and 0.476% for N, 0.0483% and 0.0676% for K, and 7.5 and 12.75 meq/l for Mg in the initial and subsequent seasons, respectively. Moreover, the leaf N and K contents at the lowest PS and vinasse concentrations were found to be 2.35% and 1.93% for N, and 3.25% and 3.00% for K in the initial and subsequent seasons, respectively, which were insufficient to meet the growth requirements of sweet pepper plants. This observation aligns with a previous findings^57^ suggested that a provisional adequate range of 3.65–3.75% for N and 3.4–3.5% for K in leaves (measured at the first flowering stage) is necessary for optimal sweet pepper plant growth and yield. Below this range, symptoms of deficiency may manifest. Concurrently, the appropriate application of vinasse, with its optimal acidity level for nutrient availability in soils (around pH 6.5–7) (Table 2), supplied essential nutrients for chlorophyll synthesis. This subsequently may increase stomatal conductance, higher photosynthetic activity rates, and carbohydrate accumulation^58^, culminating in enhanced plant biomass accumulation and increased leaf area^59^.

**Table 1:** Soil chemical and physical properties of the experimental soil, in the growing seasons of 2018/2019 and 2019/2020.

Soil properties		Seasons	
		2018/2019	2019/2020
Chemical properties	EC* (dS/m)	3.50	4.43
	pH	7.78	7.57
	N (%)	0.462	0.476
	P (%)	0.619	0.655
	K (%)	0.0483	0.0676
	HCO_3_^-^ (meq/l)	3	2.5
	Ca^++^ (meq/l)	28.5	41
	Mg^++^ (meq/l)	7.5	12.75
	Cl^-^ (meq/l)	9	15.75
	SO_4_^--^ (meq/l)	23	25.8125
Physical properties	Sand (%)	67	70
	Silt (%)	11	8
	Clay (%)	22	22
	Textures	Sandy clay loam	Sandy clay loam

* E.C.: Electrical conductivity.

**Table 2:** Physical and chemical properties of vinasse, in the two growing seasons of 2018/2019 and 2019/2020.

Vinasse properties	Seasons	
	2018/2019	2019/2020
Density (kg/m^3^)	1.26	1.20
pH	6.8	6.8
N (%)	2	2
P (%)	0.192	0.187
K (%)	3.2	3
Mg (%)	1.34	1.30
Fe (%)	0.080	0.085
Ca (%)	0.072	0.070
Zn (%)	0.05	0.05
Mn (%)	0.020	0.025

The presence of Si in the cell wall of xylem vessels is responsible for the beneficial impacts observed in plant biomass and growth characteristics when using PS application. This deposition prevents vessels from collapsing under high transpiration rates caused by elevated summer temperatures (Fig. 1) throughout the season^60^. Furthermore, foliar Si application creates a physical barrier beneath the leaf cuticle, effectively reducing transpiration rates by approximately 30%^61^. Moreover, supplying adequate Si nutrition, particularly at 0.5 g/l PS, could influence cell wall stiffness, durability, and flexibility, which subsequently affects plant architecture. This improvement results in enhanced leaf erectness, greater interception of solar radiation, and overall plant vigor^62^. However, increasing PS to 1 g/l led to decrease vegetative characteristics, possibly due to elevated Si levels being perceived as stress by pepper plant. In response, pepper plant might increase the production of non-essential compounds that negatively impact plant metabolism and growth^63^. Excessive Si concentrations could potentially hinder the absorption of other essential nutrients and disrupt plant metabolic processes. For instance, sunflowers subjected to PS substrate drenches containing 100 and 200 mg/l of Si exhibited growth abnormalities^64^. Generally, accumulator plants are less sensitive to excess Si compared to non-accumulator plants like pepper plant^13^.

Furthermore, the increased plant biomass observed in the second season could be attributed to the lignification of roots and stems in plants that have been growing for over a year^65^. Conversely, the decrease in leaf area during the second year might be explained by the suppressive impact of aging on biosynthesis-related enzymes in plant that have been growing for more than a year^66^. Prior research has reported significant enhancements in the fresh and dry biomass of pepper fruits subjected to vinasse^67^. Spraying sweet pepper plants with PS was found to maintain higher leaf areas^68^. This enhancement was attributed to elevated levels of chlorophyll, overall free amino acids, and TSS in pepper leaves and stems due to Si, subsequently stimulating increased photosynthetic rates through improved gas exchange within leaves and alterations in basic chemical processes within plant cells, thereby fostering biomass increase^69^.

The positive effects of vinasse and PS on the fruit yield potential of sweet pepper can be attributed to several factors. Firstly, vinasse improved soil health due to its rich composition of various micronutrients and macronutrients as shown in (Table 2), and growth regulators^58^, leading to improved access to and utilization of essential nutrients, along with their translocation within the plant. Furthermore, the presence of Si in PS plays a role in enhancing fruit yield potential. Silicon reduces water loss through cuticular transpiration and enhances cell wall flexibility while the plant is growing. This is achieved by its interaction with pectins and polyphenols^70^. This leads to increased ability to withstand physical forces and improved exposure to sunlight for effective photosynthesis^71^. Moreover, Si reduces plant transpiration rate, thereby enhancing water use efficiency. This, in turn, promotes various physiological processes, improves plant nutrient uptake from vinasse (Table 2), and increases the rate of photosynthesis^72^. These factors contribute to improved vegetative growth by facilitating the translocation of assimilates into the reproductive parts of the plant, ultimately resulting in higher fruit yield potential^73^. However, the improvement in relative yield in the second season as opposed to the first year could be credited to the declining performance of the control treatment (0 g/l of PS and 0 l/m^3^ of vinasse) in terms of vegetative growth, flower formation, and higher yield production. Consequently, the treated plants showed a greater positive response to the higher dosage treatments, leading to an increase in relative yield in the second season.

The present findings align with previously reported data^74^ which revealed that application of Si in the nutrient solution mitigated the adverse consequences of salt stress on fruit of tomato plants, resulting in higher yield and healthier fruit compared to untreated plants. Similarly,^75^ stated a substantial increase in yield, up to 34.37%, when pepper was treated with different amounts of vinasse as opposed to the control. The treatment of exogenous silicon to solanaceous plants for tomato^76^ and pepper^77^ has consistently shown to enhance development and yield, especially while grown in greenhouse conditions^78^. Specifically, pepper plant experienced an increase in yield by 8.4% when treated with silicon, as opposed to the control^79^.

These optimized results were anticipated due to the positive effect of PS; Enhanced silicon availability due to PS application promoted root development, resulting in improved nutrient uptake from the soil, facilitated by vinasse drenching, thus increasing nutrient substances in the tissue of leaves^80^. The stimulation of H-ATPase in the membranes could be the cause for the influence of Si on the absorption of potassium^81^. The appropriate concentration of PS in blending with vinasse helped maintain leaf tissue water balance, resulting in stable chloroplasts and more efficient photosynthesis, ultimately leading to higher amounts of TSS, ascorbic acid, N, and K in sweet pepper^82,83^.

Furthermore, the treatment of vinasse has positive influences on soil biological, chemical, and physical characteristics. It has a significant impact in the process of metabolizing different components, for example protein synthesis, auxins, chlorophyll, enzymes, and amino acids, contributing significantly to several metabolic processes in plants^84^. Comparative outcomes were previously noted^85^ indicating that drought-stressed sweet pepper plants treated with PS every 4 days had shown an increase in K content. Moreover,^13^ demonstrated that when 125 mg/l of silicon was applied to pepper plants, it led to an enhancement in the levels of TSS and chlorophyll a and b in both leaves and stems. The plants treated with 60 mg/l of silicon had the greatest concentration of amino acids in their leaves and roots. Our findings also align with^86^, who reported an improvement in leaf elements content in sugarcane plants with vinasse application. Furthermore, vinasse application enhanced the availability of soil phosphorus after crop harvest. Recently,^73^ found that nutrient uptake (N, P, K) and quality indicators (carbohydrates and proteins) of potato tubers were higher with recommended fertilizer doses supplemented with 2 t/ha of vinasse.

## Conclusion

The present research highlighted the long-term positive effects of combined potassium silicate (PS) foliar application and vinasse drenching application on plant growth, fruit yield, and fruit quality characteristics of sweet pepper cultivated under greenhouse conditions over two consecutive seasons. The synergistic interaction between a moderate PS concentration (0.5 g/l) and the highest vinasse concentration (3 l/m^3^) significantly enhanced chlorophyll index, N and K contents, and leaf area compared to control treatments, leading to increased plant biomass and fruit yield. These results suggested that the application of PS and vinasse positively influenced the physiological processes and nutrient content of sweet pepper plant, ultimately enhancing fruit quality. While 0.5 g/l PS concentration showed superiority across all vinasse concentrations regarding vegetative growth and fruit yield, the highest PS concentration (1 g/l) combined with 3 l/m^3^ vinasse significantly improved relative yield and fruit quality, particularly TSS and ascorbic acid content. This study pointed out to the potential of using PS and vinasse as sustainable biostimulants to enhance plant growth, productivity, and fruit quality of sweet pepper plant under greenhouse cultivation, contributing to effective agricultural management practices and offering insights into optimizing crop performance and resource efficiency in greenhouse vegetable production. Further research endeavors could delve into exploring the long-term effects of PS and vinasse and investigating their impact on other cultivars to fully comprehend their influence throughout the extended season of sweet pepper.

### Supplementary Information

Supplementary Information.

## Data Availability

All data generated or analyzed during this study is included in this published article.
