# Supplementary material for: Potassium silicate and vinasse enhance biometric characteristics of perennial sweet pepper (Capsicum annuum) under greenhouse conditions
Source: Sci Rep. 2024 May 16;14:11248. doi: 10.1038/s41598-024-61454-z (PMC11099072; doi:10.1038/s41598-024-61454-z)
Supplement: Supplementary file 1 — Supplementary Information. [file 41598_2024_61454_MOESM1_ESM.pdf]

**Potassium Silicate and Vinasse Enhance Biometric Characteristics of Perennial Sweet Pepper (*Capsicum annuum*) under Greenhouse Conditions**

**Mahmoud S. Rady, Ibrahim M. Ghoneim, Mostafa N. Feleafel, and Shimaa M. Hassan**

Shapiro-Wilk test for normality

| Season | Variable | Alpha Value | p-value | Statistic value | $H_0$ Hypothesis | Data distribution | |
|---|---|---|---|---|---|---|---|
| 2018/2019 | Plant fresh biomass | 0.05 | 0.260 | 0.963 | Fail to reject $H_0$ | Normal | 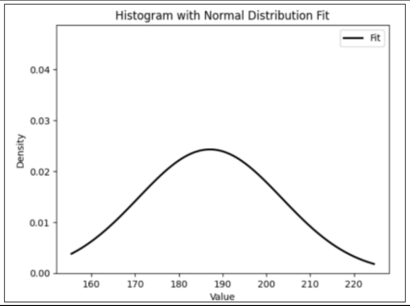  |
| | Plant dry biomass | 0.05 | 0.211 | 0.960 | Fail to reject $H_0$ | Normal | 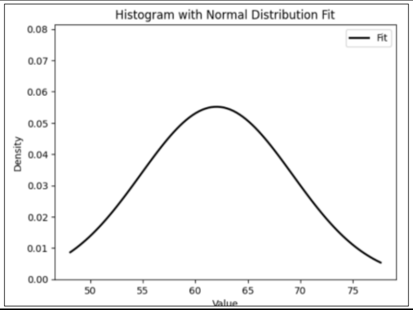  |

| | | | | | | | |
|---|---|---|---|---|---|---|---|
| | Total leaf area per plant | 0.05 | 0.268 | 0.963 | Fail to reject $H_0$ | Normal | 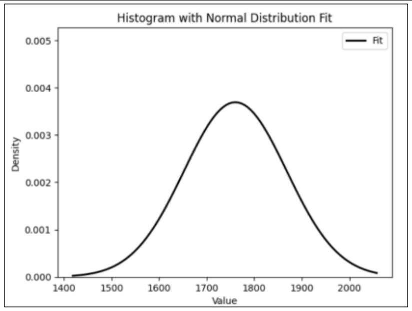 |
| | Fruit weight | 0.05 | 0.133 | 0.953 | Fail to reject $H_0$ | Normal | 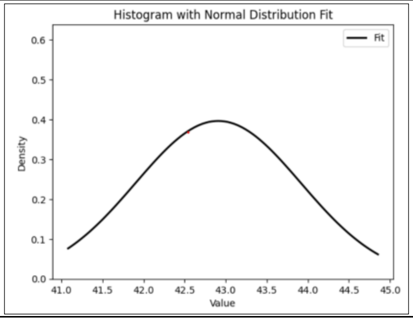 |
| | Total yield per $m^2$ | 0.05 | 0.216 | 0.960 | Fail to reject $H_0$ | Normal | 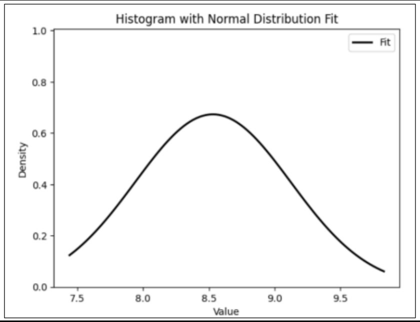 |
| | Chlorophyll index | 0.05 | 0.559 | 0.974 | Fail to reject $H_0$ | Normal | 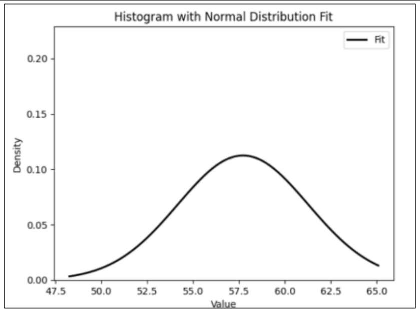 |

| | | | | | | | |
|---|---|---|---|---|---|---|---|
| | Leaves' N content | 0.05 | 0.116 | 0.951 | Fail to reject $H_0$ | Normal | 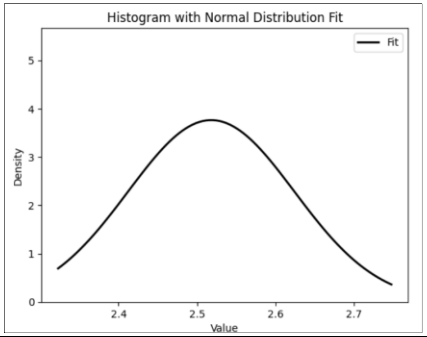  |
| | Leaves' K content | 0.05 | 0.284 | 0.964 | Fail to reject $H_0$ | Normal | 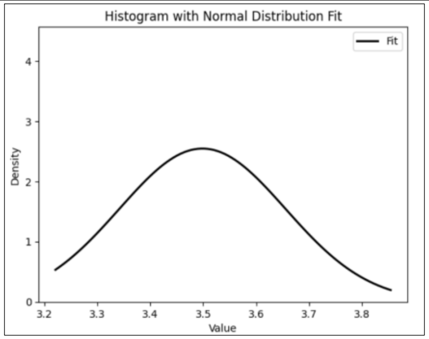  |
| | Fruits' TSS | 0.05 | 0.188 | 0.958 | Fail to reject $H_0$ | Normal | 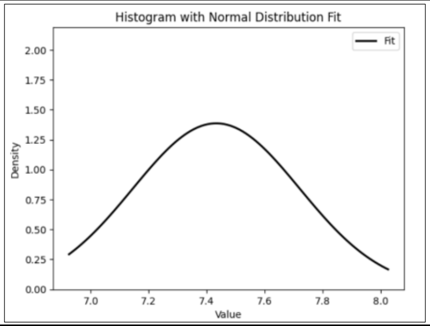  |
| | Ascorbic acid content | 0.05 | 0.152 | 0.955 | Fail to reject $H_0$ | Normal | 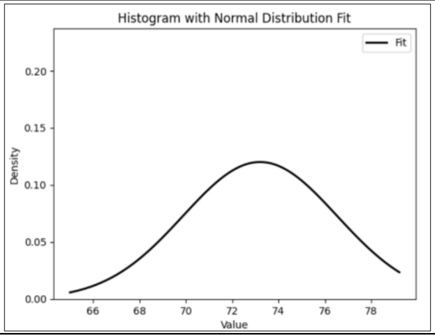  |

| | | | | | | | |
|---|---|---|---|---|---|---|---|
| 2019/2020 | Plant Fresh Biomass | 0.05 | 0.211 | 0.960 | Fail to reject $H_0$ | Normal | 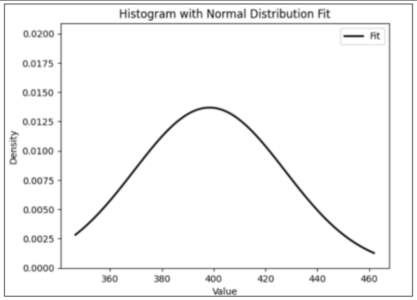 |
| | Plant Dry Biomass | 0.05 | 0.250 | 0.962 | Fail to reject $H_0$ | Normal | 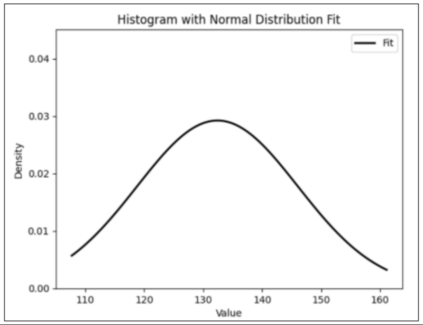 |
| | Total leaf area per plant | 0.05 | 0.185 | 0.958 | Fail to reject $H_0$ | Normal | 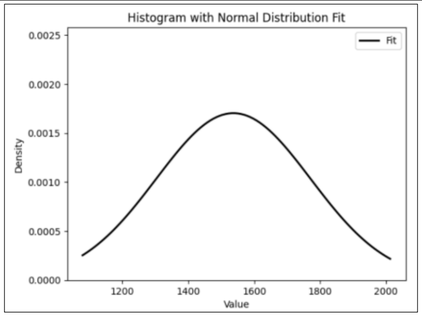 |
| | Fruit weight | 0.05 | 0.123 | 0.952 | Fail to reject $H_0$ | Normal | 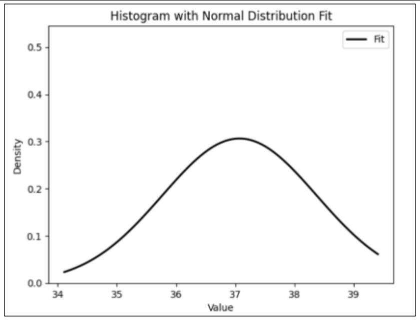 |

| | | | | | | | |
|---|---|---|---|---|---|---|---|
| | Total yield per $m^2$ | 0.05 | 0.210 | 0.960 | Fail to reject $H_0$ | Normal | 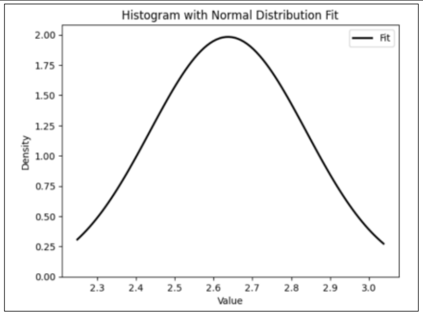 |
| | Chlorophyll index | 0.05 | 0.088 | 0.948 | Fail to reject $H_0$ | Normal | 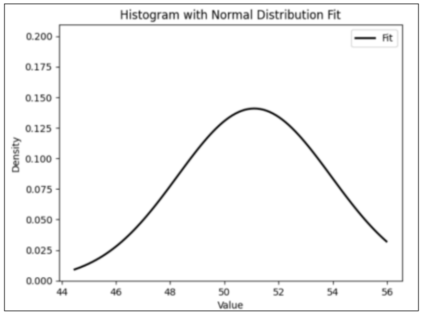 |
| | Leaves' N content | 0.05 | 0.149 | 0.955 | Fail to reject $H_0$ | Normal | 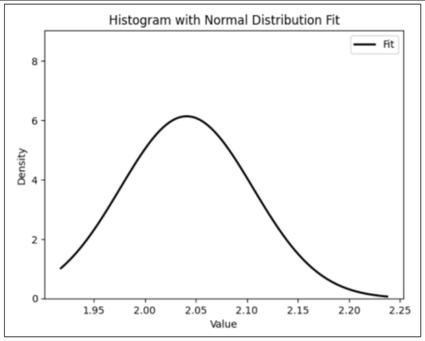 |
| | Leaves' K content | 0.05 | 0.152 | 0.955 | Fail to reject $H_0$ | Normal | 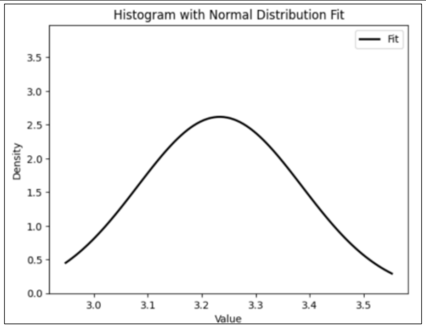 |

| | | | | | | | |
|---|---|---|---|---|---|---|---|
| | Fruits' TSS | 0.05 | 0.247 | 0.962 | Fail to reject $H_0$ | Normal | 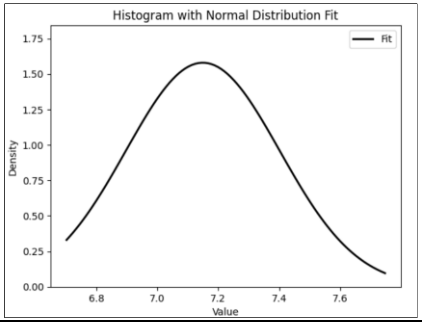 |
| | Ascorbic acid content | 0.05 | 0.072 | 0.945 | Fail to reject $H_0$ | Normal | 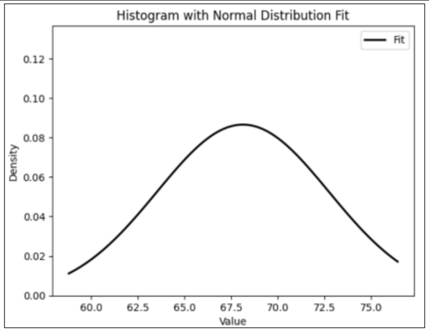 |
